# Tunable Bandpass Filtering in Coupled Nanodrums Enabled by 1:1 Internal Resonance

**DOI:** 10.3390/mi17030379

**Published:** 2026-03-20

**Authors:** Yikun Liu, Jiaxin Miao, Haoran Wang, Jinghong Tang, Cao Xia, Xiaoyu Liu

**Affiliations:** School of Mechanical Engineering, Sichuan University, Chengdu 610065, China

**Keywords:** nanoelectromechanical resonator, radio frequency (RF), 1:1 internal resonance, high tunability, two-dimensional materials

## Abstract

In recent years, microelectromechanical systems (MEMS) filters exploiting structural nonlinearity and coupled resonance have enabled programmable passband shaping beyond traditional single-peak designs, yet they still face low operating frequencies and limited electrical tuning range. Here, leveraging 1:1 internal resonance, we propose a gate-programmable tuning strategy for two-dimensional (2D) material-based nanoelectromechanical systems (NEMS), enabling high-frequency operation and wide-range reconfigurability. Benefiting from the high resonant frequency and wide electrostatic tunability of 2D materials such as MoS_2_, our theoretical analysis indicates wide-range programmability up to $f/f_{0}\approx 200\%$. Sweeping $V_{g1}=V_{g2}$ from 9 to 16 V while maintaining ≈1:1 frequency matching shifts the passband upward quasi-linearly at 4.4~MHz/V. In contrast, with the coupling strength nearly unchanged, mV-level bias mismatch perturbs the frequency ratio by $10^{-5}$, enabling highly sensitive bandwidth trimming from 3.18 to 5.20 kHz, supporting a two-step strategy of coarse center-frequency tuning followed by fine bandwidth control. To broaden the bandwidth, we further analyze a three-drum case: with $V_{g1}=V_{g2}=V_{g3}=16\;V$, the bandwidth reaches 21.79 kHz with a 5056.05 dB/MHz transition slope and 0.95 dB ripple, which is nearly 4 times wider than the two drum case with the same gate voltage. This study shows that 1:1 internal resonance can be used to tune the bandpass response of NEMS resonators. All results are obtained from theoretical modeling and numerical simulations.

## 1. Introduction

The growing demand for radio-frequency (RF) communication devices has driven the development of filters with low power consumption, high selectivity, and compact size. Traditional electrical filters, such as pure capacitor/inductor networks or active filters, are mature in system integration, but their frequency stability is inevitably affected by temperature coefficients, device aging, and electromagnetic environment disturbances [1,2]. In contrast, resonance-based filtering in Micro/Nanoelectromechanical Systems (MEMS/NEMS) exploits the natural resonances of the devices, offering intrinsically high selectivity and sharp skirts. Their chip-scale form factor enables compact integration in RF front ends, and their resonance-based operation provides strong immunity to electromagnetic interference and improved frequency stability, supporting reliable and repeatable performance when co-integrated with on-chip circuitry. Moreover, progress in electrostatic frequency tuning of resonators in MEMS/NEMS over the past two decades has opened new possibilities for the design of high-performance mechanical filters [3,4,5,6,7,8]. In parallel, recent advances in reconfigurable filtering and spectral-response devices on emerging integrated platforms have further underscored the demand for compact, tunable, and highly selective signal-processing components [9]. Together, these developments provide a broader context and strong motivation for exploring mechanically programmable filtering concepts in MEMS/NEMS resonators.

In the typical research on MEMS tunable mechanical filters, for instance, the Jian Zhao team utilized two micrometer beams with electromagnetic coupling to fabricate a high-performance two-stage bandpass filter based on the dynamic snap-through phenomenon, with its center frequency tunable within the range of 23.81 kHz to 25.16 kHz [5]; Saad llyas designed an H-shaped resonator composed of two clamped-clamped microbeams, achieving broadband tunability through combined resonance generated by mechanical amplification and mixed frequency excitation, and capable of simultaneously up-conversion and down-conversion in multiple frequency bands. By adjusting the gate voltage bias $V_{DC,MC}$ from 0 to 16 V, the center frequency could reach 109 to 115 kHz [7]. Although these works demonstrated the tunable filtering paradigm of “structural nonlinearity + coupled resonance”, overall, they still faced issues such as low operating frequency and limited continuous tuning range. Therefore, in recent years, researchers have gradually turned their attention to higher resonant frequencies and more easily tuning two-dimensional NEMS resonant filters and other fields.

It is worth noting that recent studies have shown that two-dimensional (2D) material NEMS resonators provide a promising platform for tunable high-frequency operation, owing to their relatively high resonant frequencies (typically in the tens of MHz range) and wide electrical tunability [10,11,12,13,14]. For example, Philip X.-L. Feng’s team demonstrated direct electrostatic gate tuning in MoS_2_–graphene van der Waals heterostructure drumhead resonators, continuously tuning the same device, and mode from ∼23 to ∼107 MHz, and achieving up to $\Delta f/f_{0}\approx 370\%$ [10]. They also studied FePS3 drumhead resonators under electrostatic gate bias, identifying three types of frequency–voltage tuning curves and reporting a maximum fractional tuning range of $\Delta f/f_{0}=32\%$, with resonance frequencies in the range of ∼10–50 MHz [11]. For comparison, monolayer graphene resonators can exhibit even larger electrostatic tunability. Chen et al. reported that, in the lowest built-in-tension limit, a tuning range approaching $∆f/f_{0}∼300\%$ can be achieved [12,13]. In addition to electrostatic tuning, an on-chip strain-engineering approach has been demonstrated, where a silicon-based comb-drive MEMS actuator applies a controllable in-plane tensile strain to an integrated 2D-material NEMS resonator (primarily multilayer graphene), enabling continuous and wide-range tuning of the intrinsic resonance frequency, with a maximum fractional tuning of ∼75% [15].

On the other hand, two-dimensional (2D) material NEMS resonators, owing to their ultralow mass and membrane-type mechanics, readily exhibit rich nonlinear dynamics under electrostatic actuation, including Duffing-type stiffness nonlinearity, nonlinear dissipation, and intermodal nonlinear coupling [16,17,18,19,20,21,22]. For instance, Castellanos-Gomez’s group reported an asymmetric, frequency-stiffening nonlinear resonance in a 2 µm-diameter monolayer MoS_2_ resonator when the drive power exceeded −9 dBm [17]. Davidovikj et al. further demonstrated that ultrathin 2D-material drum resonators enter a pronounced Duffing nonlinear regime already at piconewton-level electrostatic drives, and leveraged the nonlinear frequency response to quantitatively extract Young’s modulus [18]. Akita’s group observed hardening-type Duffing nonlinearity in a graphene drum resonator as the actuation laser power $P_{\mathrm{act}}$ was increased from 296 to 736 µW, and showed that the nonlinearity can be tuned via Fabry–Perot–enhanced photothermal backaction from the probe laser [19].

Such nonlinearities provide a natural route to internal resonance in coupled nonlinear systems: when the natural frequencies of two modes approach an integer ratio (e.g., 1:1, 1:2, 1:3), efficient energy exchange and response redistribution may occur between modes [20]. In 2D resonators, this mechanism has been directly observed. Naik’s group drove a few-layer MoS_2_ drum into the nonlinear regime and reported clear intermodal internal resonances (1:2 and 1:1) in an atomically thin mechanical system [21]. Moreover, the mode interaction strength itself can be electrostatically programmed; Prasad, Arora, and Naik tuned the cooperativity between different MoS_2_ vibrational modes by over an order of magnitude using a DC gate voltage $V_{g}^{dc}$ [22]. Importantly, internal-resonance–induced energy redistribution can transform a single peak-like response into a flat-top passband and produce a steeper roll-off via enhanced out-of-band suppression, as demonstrated in MEMS bandpass filters exploiting internal resonance [23,24]. However, to the best of our knowledge, internal-resonance-based NEMS filter implementations and systematic gate-programmable studies remain largely unexplored.

Collectively, these results motivate the use of tunable internal-resonance dynamics in 2D NEMS as a compact and reconfigurable platform for next-generation miniaturized mechanical filters. In addition, 2D material resonators are intrinsically compact and often exhibit stronger nonlinearities at the nanoscale, and recent studies have further demonstrated their potential for mechanical signal transfer between spatially separated nanodrums [25], thereby providing a material and device foundation for next-generation miniaturized, tunable, and high-performance mechanical filters.

Therefore, by introducing 1:1 internal resonance as the core mechanism for shaping the filter curve into the two-dimensional material NEMS resonators, it is expected to further obtain a quasi-rectangular flat-top bandpass response on top of its MHz-level natural frequency and excellent tunability, providing a promising route to design NEMS mechanical RF filters. In this work, we consider two spatially separated nanodrums based on MoS_2_ flakes and investigate, through gate-voltage tuning of their natural frequencies and coupling strength, programmable control of the center frequency and bandwidth. Furthermore, we investigate the impact of weak nonlinear effective stiffness on the programmed center-frequency shift in the small-amplitude regime and extend the system to a three-drum coupled model, achieving an approximately fourfold bandwidth expansion.

## 2. Device Platform and Methods

### 2.1. Design of Nano Drums

**Figure 1:**
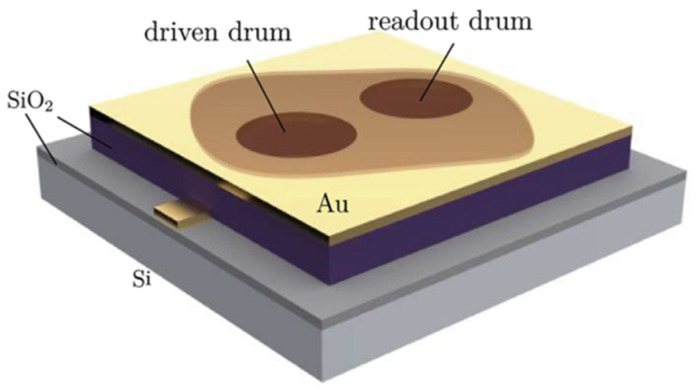
Conceptual schematic of the proposed dual-nanodrum device layout.

**Figure 2:**
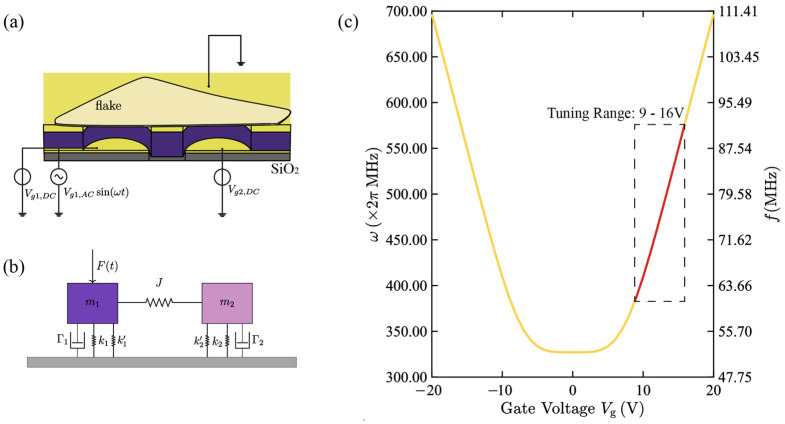
(**a**) Schematic diagram of the device cross-sectional structure; (**b**) Equivalent coupling model of the dual resonator system, where $m_{1}$ and $m_{2}$ represent the equivalent masses of the two drums, J is the coupling strength, k is the equivalent linear stiffness, and k’ represents the nonlinear stiffness term; (**c**) Curve showing the relationship between the membrane natural frequency and the gate DC bias within the tuning range of 9 V to 16 V. Within the gate voltage tuning range of 9 V to 16 V, the natural frequency shows an approximately linear relationship with the gate voltage, and the linear working range is marked in red. Numerically calculated relationship between the membrane natural frequency and the gate DC bias in the adopted tuning range.

We have demonstrated that by using a spatially separated dual-resonator drum structure, under appropriate damping conditions and gate voltage configuration, effective filtering of RF signals can be achieved. The device structure is shown in Figure 1: As a possible fabrication route for such a structure, an SiO_2_ layer is first formed on the Si substrate, followed by deposition and patterning of the bottom Au electrode. After that, an additional SiO_2_ insulating layer with a thickness of approximately 300 nm is deposited by PECVD to cover the Au electrode; then, the top electrode is deposited and patterned, followed by etching to define the cavity structure. Subsequently, through a dry transfer process, the MoS_2_ thin flake obtained by mechanical exfoliation is precisely transferred above the cavity, allowing the same flake to simultaneously span over two cavities and naturally form two independent suspended circular membrane structures, namely the dual-resonator drum system. The overall appearance and layered configuration of the device are illustrated in Figure 1, while a more detailed cross-sectional view is provided in Figure 2a.

On the two Au electrode terminals leading out, a DC voltage is applied, respectively, to provide independent gate biases $V_{g1}$ and $V_{g2}$ for driving the drum and reading the drum. Through the action of capacitive electrostatic force, the inherent resonant frequency of the membrane and the coupling strength between the two drums can be continuously controlled, thereby achieving electrical tunability of the filter center frequency and bandwidth. On the DC gate bias of the driving drum, an AC voltage with the same frequency as the input signal is further superimposed. For clarity, a single-tone input $V_{g,\mathrm{AC}}\cos\left(\omega t\right)$ is considered, the electrostatic driving force induced by this AC signal excites the deflection vibration of the driving drum and transfers the energy effectively to the reading drum through a 1:1 internal resonance mechanism, thereby inducing its synchronous vibration.

The mechanical response of the readout drum is converted into an electrical signal and detected using electrical readout schemes such as dual-source mixing or down-conversion (Due to the measurement bandwidth limitation, it can typically cover up to several hundred MHz). By setting a threshold criterion for the amplitude at the reading end, selective passage of signals in the target frequency band can be achieved, thereby filtering out spurious signals at non-target frequencies.

### 2.2. Coupling Model

As shown in Figure 2a, for a single-layer MoS_2_ drum, we adopt a membrane model and consider its first-order modal shape $\xi \left(a\right)=x\left(1-\frac{a^{2}}{r^{2}}\right),\;a\in \left[0,r\right]$. The total potential energy $U_{\mathrm{el}}+U_{\mathrm{es}}$ under an applied DC gate bias $V_{g}$ is evaluated, where $U_{\mathrm{el}}$ denotes the membrane’s elastic energy and $U_{\mathrm{es}}$ denotes the capacitive electrostatic potential energy. The first-order equivalent linear stiffness k is given by the second derivative of $U_{\mathrm{el}}+U_{\mathrm{es}}$ with respect to the amplitude x, evaluated at the equilibrium $x=x_{g}$. Together with the effective mass $m_{\mathrm{eff}}$, the natural angular frequency of the drum is expressed as (see Supplementary Materials for the detailed derivation).

(1) $$\omega_{0}=\sqrt{\frac{k}{m_{\mathrm{eff}}}}=\sqrt{\frac{\pi Eh/\left(1-v^{2}\right)\;\left(8x_{g}^{2}/r^{2}+2\varepsilon_{0}\right)-\varepsilon_{0}\pi r_{g}^{2}V_{g}^{2}/{\left(x_{c}-x_{g}\right)}^{3}}{\alpha \rho h\pi r^{2}}}$$

Among them, *E*, *v*, ρ and *h* represent the Young’s modulus, Poisson’s ratio, density and thickness of the membrane material, $x_{g}$ represents the central displacement at the static equilibrium position, $x_{c}$ is the initial electrode gap, $r_{g}$ is the effective grid radius, and α is the quality correction coefficient determined by the modal shape. The tuning relationship of the membrane’s natural frequency with respect to the gate’s DC bias is shown in Figure 2c.

Furthermore, we establish a force-electric coupling model for the double drum system, as shown in Figure 2b. Based on this, we derived the normalized coupled Equation (2):

(2) $$\left\{\begin{array}{c} {\ddot{x}}_{1}+\gamma_{1}{\dot{x}}_{1}+\omega_{1}^{2}x_{1}=j_{11}x_{2}+j_{12}x_{c}x_{2}^{2}+j_{13}x_{c}^{2}x_{2}^{3}+\alpha_{11}x_{1}+\alpha_{12}x_{c}x_{1}^{2}+\alpha_{13}x_{c}^{2}x_{1}^{3}+f_{d}\cos\left(\omega_{d}t\right)\\ {\ddot{x}}_{2}+\gamma_{2}{\dot{x}}_{2}+\omega_{2}^{2}x_{2}=j_{21}x_{1}+j_{22}x_{c}x_{1}^{2}+j_{23}x_{c}^{2}x_{1}^{3}+\alpha_{21}x_{2}+\alpha_{22}x_{c}x_{2}^{2}+\alpha_{23}x_{c}^{2}x_{2}^{3} \end{array}\right.$$

Here, $x_{1}$ and $x_{2}$ represent the displacements of the driven drum and readout drum membranes respectively. $\gamma_{1}$ is the normalized damping, with a value of 1/Q = 3.66 × $10^{-5}$. $\omega_{i},j_{ij}$ are the normalized natural frequencies and coupling coefficients, and $f_{d}\cos\left(\omega_{d}t\right)$ represents the AC drive applied to the gate of the driving drum.

By expanding the coupled capacitor energy with respect to the membrane displacement, the equivalent coupling force form of the driving drum (drum 1) to the readout drum (drum 2) can be obtained as follows:

(3) $$F_{2,\mathrm{AC}}=J_{21}x_{1}\left(t\right)+J_{22}x_{1}^{2}+J_{23}x_{1}^{3}$$

and the effective nonlinear restoring term of drum 2 is expressed as

(4) $$k^{\prime }x_{2}=\alpha_{21}x_{2}+\alpha_{22}x_{2}^{2}+\alpha_{23}x_{2}^{3}$$

The coupling coefficients and nonlinear stiffness coefficients take the form

(5) $$J_{2i}=-\lambda^{2}\frac{V_{g,1}V_{g,2}}{2C_{m}}\frac{1}{\left(i+1\right)x_{c}^{i-1}}\left(i\geq 1\right)$$

(6) $$\alpha_{2i}=-\lambda^{2}\frac{V_{g,2}^{2}}{2C_{m}}\frac{1}{\left(i+1\right)x_{c}^{i-1}}\;\left(i\geq 1\right)$$

where $\lambda =\varepsilon_{0}\pi r^{2}/x_{c}^{2}$ and $C_{m}$ is the equivalent coupling capacitance (set to 0.5 pF). Since the natural frequency $\omega_{0}$ and coupling strength $J_{ij}$ of the single drum is explicitly dependent on the gate DC bias voltage, it is possible to achieve electro-acoustic tuning of the system’s central frequency and bandwidth by choosing appropriate $V_{g,1}$ and $V_{g,2}$.

It should be noted that when adjusting the gate DC bias, both the natural frequency and coupling strength will change simultaneously, which limits the ability to independently control both. However, by selecting an appropriate number of two-dimensional material membranes, the inherent frequency and the sensitivity of the coupling strength to the gate voltage can be effectively changed, thereby achieving approximate independent tuning to a certain extent. Additionally, introducing additional electrodes or structural degrees of freedom can further improve this issue. The relevant solutions will be discussed in detail in the subsequent work.

## 3. Filtering Principles and Programming Strategies

### 3.1. Internal-Resonance–Enabled Bandpass Formation

For two resonators with nearly identical natural frequencies, efficient energy exchange can occur between the modes, thereby exciting 1:1 internal resonance and leading to the phenomenon of linear mode splitting. When the coupling strength and damping coefficient satisfy j/γ ≥ ω_0_, a typical bimodal amplitude-frequency curve is formed. Further, by adjusting the appropriate coupling strength and damping ratio to the critical state where j/γ ≈ ω_0_, the originally split resonant peaks gradually broaden and overlap, resulting in a rectangular-like amplitude-frequency curve.

As shown in Equation (2), this double-resonator system can be regarded as a linearly coupled oscillator system modified by weak nonlinear terms. Under 1:1 internal resonance conditions, the system dynamics are mainly dominated by odd-order coupling terms, among which the linear coupling terms play a decisive role in energy exchange and mode splitting, while the cubic nonlinear term only modifies the response characteristics.

Within this working range, the frequency response of the system can be understood as the linear mode splitting behavior enhanced by internal resonance. Therefore, the peak response frequency of the system is generally still distributed near the linear mode, but is modulated by nonlinear effects, and the specific offset depends on parameters such as vibration amplitude, coupling strength, and external driving conditions.

**Figure 3:**
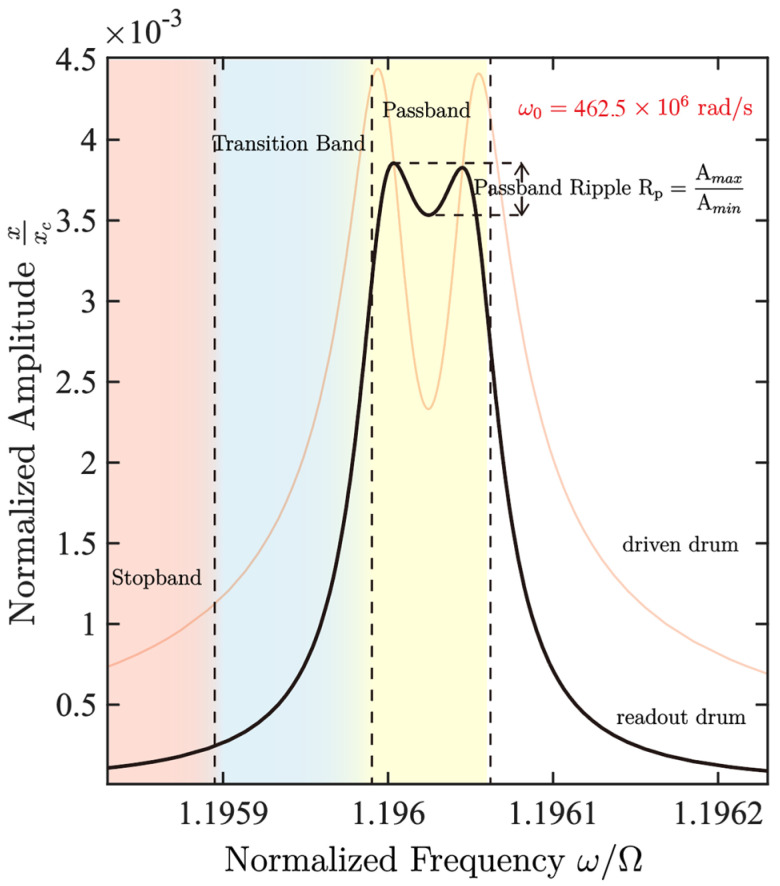
Frequency response curve when $V_{g1}=V_{g2}=12\;V$. The black curve represents the frequency response of the read drum, and the red curve represents the frequency response of the drive (input) drum. Different background colors are used in the figure to indicate the passband, transition band, and stopband regions.

For the representative operating condition considered in this subsection ($V_{g1}=V_{g2}=12\;V$), the numerical values of the main coefficients in Equation (2) are taken as $\Omega =4.6245\times 10^{8}\;\mathrm{rad}/s$; except for Ω, all the remaining coefficients listed below are normalized parameters: $\omega_{1}=\omega_{2}=1,$ ${\;\gamma }_{1}=\gamma_{2}=3.0570\times 10^{-5},$ $\;j_{11}=j_{21}=4.6281\times 10^{-5},$ $\;j_{12}=j_{22}=3.0854\times 10^{-5},$ ${\;j}_{13}=j_{23}=2.3141\times 10^{-5},$ ${\;\alpha }_{11}=\alpha_{21}=4.6281\times 10^{-5},$ ${\;\alpha }_{12}=\alpha_{22}=3.0854\times 10^{-5}$, ${\;\alpha }_{13}=\alpha_{23}=2.3141\times 10^{-5}$, and $f_{d}=2.3380\times 10^{-7}$. These values are calculated from the analytical expressions derived in Section 2 and Supplementary S2 using the adopted material parameters and structural dimensions. Using the above parameter set, the amplitude-frequency curve of the readout resonator is obtained as shown in Figure 3, where ripple = 0.76 dB, roll-off slope = 4321.96 dB/MHz, and bandwidth = 4.62 kHz; it can be seen that the double-resonator filtering structure based on internal resonance has significant advantages in both passband flatness and transition band steepness.

**Figure 4:**
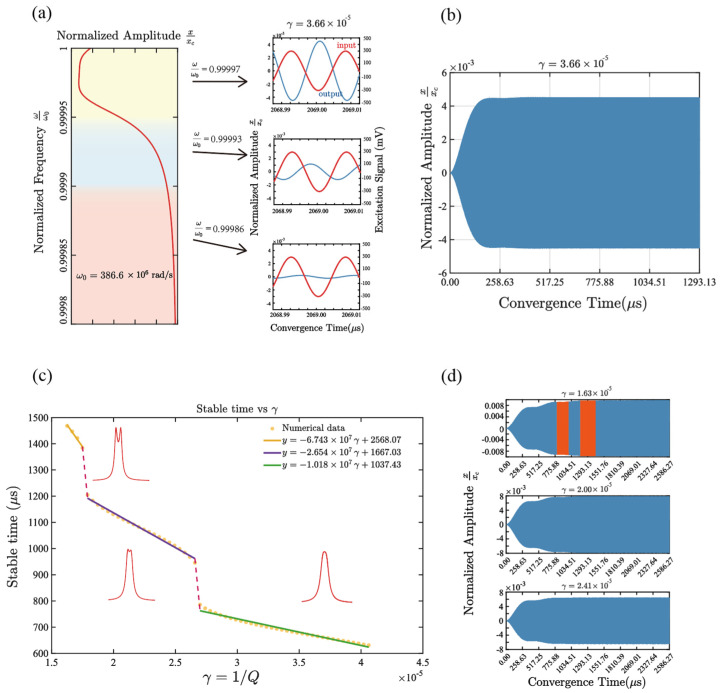
(**a**) Under the condition of $V_{g1}=V_{g2}=9\;V$ and normalized damping coefficient $\gamma =2.03\times 10^{-5}$, the schematic illustration of the filtering response corresponding to different frequency regions. For the same input excitation signal $\cos\left(0.99997\omega t\right)$, when the driving frequency is located in the passband (yellow area), the transition band (red area), or the stopband (blue area), the output response characteristics of the drum show significantly different characteristics; (**b**) An example of the process of the system vibration response evolving over time and gradually converging to the steady state, with the convergence time quantitatively extracted by the envelope criterion; (**c**) Typical time-domain responses under different damping conditions $\gamma =1.63\times 10^{-5},\;2.82\times 10^{-5}$, and $4.06\times 10^{-5}$, used to compare the influence of damping on the system convergence process; (**d**) The relationship between the system convergence time and the normalized damping coefficient under the condition of keeping $V_{g1}=V_{g2}=9\;V$ unchanged.

Figure 4a illustrates the filtering mechanism through the amplitude–frequency response of the coupled drums. With the DC gate biases set to enforce a near 1:1 condition, the two resonant frequencies are nearly equal, i.e., $\omega_{1}\approx \omega_{2}$. In this near-commensurate regime, nonlinear intermodal coupling enables efficient energy exchange only within a finite detuning window around the center frequency $\omega_{0}$. As a result, the steady-state amplitude remains high and relatively flat in this region, forming the passband. Outside this window, the intermodal interaction becomes inefficient, and the steady-state response drops sharply (stopband), thereby producing a band-pass characteristic centered at $\omega_{0}$. In operation, an AC drive at the same frequency as the input signal is applied to the driving gate, and the output is obtained by reading out the drum’s steady-state vibration amplitude, which realizes the filtering function.

It should be noted that the amplitude-frequency characteristic essentially reflects the steady-state response of the system under slow-flow modulation. Therefore, the filtering result is not determined by the instantaneous response but depends on the process by which the system converges to a stable vibration solution after the parameter setting. In practical work, after each adjustment of the gate voltage, the system needs to go through a finite transition process to reach the new steady state, and its response speed is directly determined by the convergence time. For the system shown in Figure 4b, the numerical results indicate that it takes approximately 0.7 ms for the system to reach stability after a parameter change.

To quantitatively determine the convergence time of the system, we apply the Hilbert transform to the vibration response y(t) of the readout drum to extract its envelope, thereby obtaining the instantaneous amplitude A(t). The steady-state amplitude $A_{\infty }$ is estimated as the median value of the envelope amplitude over the final 5% of the simulation time. This time interval is far enough from the initial transient process, and the median value statistics can effectively suppress the influence of local fluctuations and outliers. The convergence time $t_{s}$ of the system is defined as the earliest time point when the system reaches the steady state. If $t>t_{s}$, the relative deviation of the envelope amplitude from $A_{\infty }$ is satisfied as $\frac{\left|A\left(t\right)-A_{\infty }\right|}{A_{\infty }}<1$. The determined time *t_s_* is defined as the convergence time of the system.

In Figure 4d, the step-like decrease in the extracted convergence time with increasing γ is mainly caused by enhanced energy dissipation. At smaller γ, the coupled system often exhibits a pronounced transient beating stage, during which the envelope approaches the steady-state level intermittently; this long-lived transient contributes to the two orange branches. When γ becomes larger, dissipation suppresses such beating and accelerates the decay of the transient oscillations, so the response settles into the steady state more directly, and the corresponding orange branches disappear. As a result, the convergence time $t_{s}$ defined by the same envelope criterion shows an apparent discontinuous reduction.

Based on the above filtering and tuning mechanisms, we further adjusted the gate DC bias to control the natural frequency of the single drum and the coupling strength between the two drums. Within the model assumptions adopted in this paper, we systematically analyzed the tuning limit range that this structure can achieve. It should be noted that this limit only represents the boundary of the model’s applicability and does not imply that the device cannot be further tuned physically. On this basis, we focused on studying the impact of the tuning process on the key performance indicators of the filter, including the slope of the transition band, the flatness of the passband, and the bandwidth, etc. The results show that by reasonably designing the gate bias operating point, the controllable adjustment of the filter’s characteristics can be achieved within a wide frequency range, while maintaining excellent passband and transition band performance.

### 3.2. Center-Frequency Tuning

**Figure 5:**
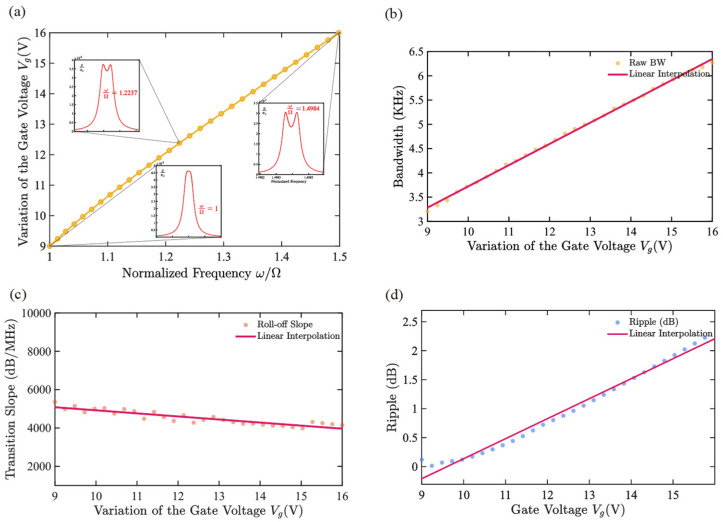
Numerically calculated center-frequency tuning characteristics of the two-resonator system under simultaneous gate-bias variation. (**a**) Schematic diagram showing the relationship of center frequency with gate voltage; (**b**–**d**) Illustrate the evolution laws of filter bandwidth, transition band slope, and passband ripple with gate voltage during the center frequency tuning process.

When both the gate DC biases $V_{g1}$ and $V_{g2}$ are increased simultaneously, while keeping the ratio of the two inherent frequencies approximately 1:1, the center frequency of the system can be continuously adjusted, thereby achieving an overall shift tuning of the center frequency, as shown in Figure 5. Considering the linear working range of the equivalent stiffness with the gate bias variation (see Figure 2c), and assuming $x_{g}\ll x_{c}$, this paper selects the gate voltage tuning range to be 9–16 V. Within this range, the center frequency moves approximately linearly towards higher frequencies with the gate voltage, and its tuning rate is approximately 4.4 MHz/V, corresponding to the maximum relative change of $\frac{\Delta \omega_{0}}{\omega_{0}}\approx 50\%$ as shown in Figure 5a.

When the center frequency moves towards higher frequencies, due to j/(γω_0_) > 1 and increasing approximately linearly with the gate voltage, the system gradually deviates from the critical coupling state, and the amplitude-frequency response tends to exhibit a typical modal splitting double-peak structure. This change leads to a decrease in the flatness of the passband and a slight reduction in the slope of the transition band.

As shown in Figure 5b–d, the change in coupling dimension caused by altering the center frequency leads to the shifting of the double peaks in the amplitude-frequency curve to both sides, causing the bandwidth to approximately linearly increase at a rate of 0.4376 kHz/V; at the same time, the passband ripple increases at a rate of 0.34 dB/V, and the slope of the transition band increases at a rate of 159.8 dB/(MHz·V).

The coefficient $J_{2i}$ is proportional to ${1/C}_{m}$. By contrast, the natural frequency $\omega \left(V_{g}\right)$ of a single drum is approximately linear with $V_{g}$ in the range of 9–16 V and does not depend on $C_{m}$. Therefore, by choosing appropriate membrane materials or geometric parameters, it is possible to achieve that $J_{ij}$ remains almost unchanged when $\omega \left(V_{g}\right)$ is changed, thereby approximately independently tuning the center frequency and coupling strength to a certain extent.

### 3.3. Bandwidth Tuning with Fixed J

**Figure 6:**
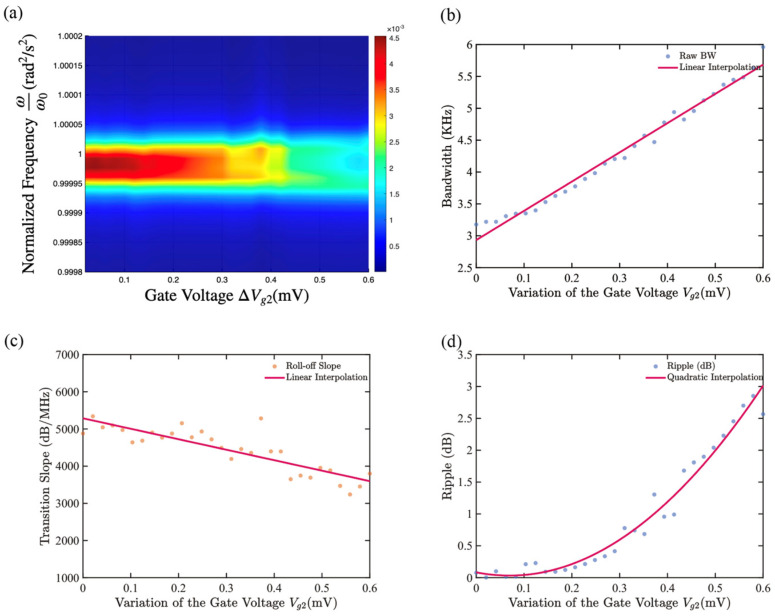
Under the condition where the coupling strength remains constant, the filtering characteristics of frequency-ratio tuning are numerically obtained by fine-tuning the gate DC bias. (**a**) When a small DC voltage perturbation is applied near $V_{g1}=V_{g2}=9\;V$, the three-dimensional cloud diagram of the amplitude-frequency response shows the continuous evolution behavior of the filter’s amplitude-frequency curve under different $V_{g2}$ conditions; (**b**–**d**) The relationships of the filter’s bandwidth, transition band slope, and passband ripple with the gate voltage fine-tuning are respectively presented during the frequency ratio tuning process.

As shown in Figure 6a, first, the gate biases of the driving drum and the reading drum are both set to 9 V. At this point, the coupling strength remains unchanged. A DC voltage perturbation of the mV level is added to the gate biases of the driving drum and the reading drum to perturb their frequency ratios by a magnitude of 0.001%. The results show that when the natural frequency of the reading drum changes to 0.0028%, the passband ripple rises to 2 dB. At this point, the amplitude-frequency response of the reading drum has significantly deviated from the ideal filtering characteristic, and the corresponding change in the gate voltage is 0.5 mV.

When the relative offset is less than 0.0028%, the amplitude-frequency response of the diaphragm shows only a slight disruption of symmetry as the detuning increases; as the frequency offset further increases, the amplitude-frequency curve gradually evolves into a more obvious bimodal structure. Within this tuning range, the slope of the transition band remains generally at 4000–5000 dB/MHz. When the frequency ratio is changed by gate bias, it gradually decreases in a trend of 2817 dB/(MHz·μV) while accompanied by certain jitter; at the same time, the filter bandwidth can be continuously adjusted within the range of 3.18 kHz–5.20 KHz and exhibits a good linear relationship, with the increase in amplitude varying approximately by 4.59 kHz/μV with the gate voltage.

Compared to the slow change in bandwidth during the center frequency tuning process, which only occurs at a rate of approximately 0.4376 kHz/V, the filter bandwidth is obviously more sensitive to the out-of-tune ratio of the two drum natural frequencies. Therefore, in practical applications, the filter center frequency can be determined first, and then the frequency ratio of the two drums can be finely adjusted to achieve highly sensitive control of the bandwidth.

## 4. Discussion

### 4.1. Center Frequency Drift Caused by Nonlinear Effective Stiffness Modulation

In fact, the nonlinear stiffness modulation term mainly plays the role of frequency shift within the current working range. Therefore, the renormalized equivalent linear stiffness term is introduced and defined as:

(7) $$\left\{\begin{array}{c} \hat{\omega_{1}^{2}}=\omega_{1}^{2}-\alpha_{11}-\alpha_{12}x_{c}x_{1}+\alpha_{13}x_{c}^{2}x_{1}^{2}\\ \hat{\omega_{2}^{2}}=\omega_{2}^{2}-\alpha_{21}-\alpha_{22}x_{c}x_{2}+\alpha_{13}x_{c}^{2}x_{2}^{2} \end{array}\right.$$

Under this definition, the normalized coupling Equation (2) can be rewritten as:

(8) $$\left\{\begin{array}{c} {\ddot{x}}_{1}+\gamma_{1}{\dot{x}}_{1}+\hat{\omega_{1}^{2}}x_{1}=j_{11}x_{2}+j_{12}x_{c}x_{2}^{2}+j_{13}x_{c}^{2}x_{2}^{3}+f_{d}\cos\left(\omega_{d}t\right)\\ {\ddot{x}}_{2}+\gamma_{2}{\dot{x}}_{2}+\hat{\omega_{2}^{2}}x_{2}=j_{21}x_{1}+j_{22}x_{c}x_{1}^{2}+j_{23}x_{c}^{2}x_{1}^{3} \end{array}\right.$$

Under the normalization adopted in this paper, the amplitude $x_{c}$ is at the order of $10^{-3}$ so the higher-order correction terms related to $x_{c}$ and $x_{c}^{2}$ are numerically significantly smaller than the constant terms. Therefore, within the present operating range, the system remains only weakly nonlinear, and the nonlinear effect mainly appears as a slight overall frequency shift rather than typical strong nonlinear behaviors such as pronounced softening, hardening, or jump phenomena.

**Figure 7:**
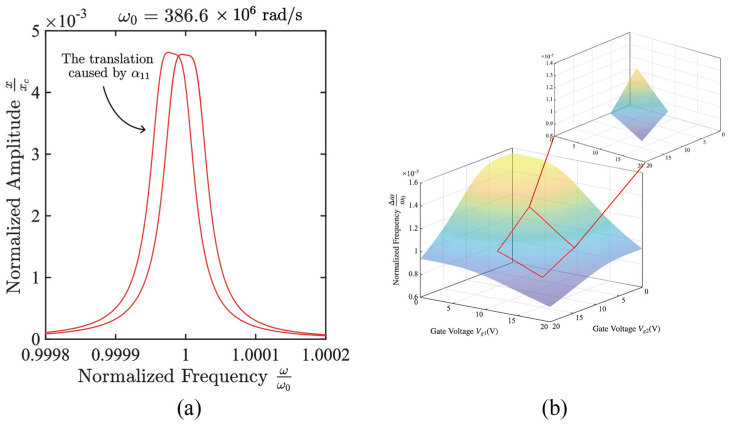
Numerically calculated influence of nonlinear stiffness modulation on the amplitude-frequency response. (**a**) Illustration of the overall shift in the amplitude-frequency response towards lower frequencies caused by the stiffness modulation term $\alpha_{11}$; (**b**) Relationship between the nonlinear stiffness coefficient $\alpha_{11}$ and the gate DC bias $V_{g1},V_{g2}$ within the range of 0 V to 20 V, where the working range of 9 V to 16 V is marked with a red box.

Thus, we may approximately write $\hat{\omega_{i}^{2}}\approx \omega_{i}^{2}-\alpha_{i1}\;\left(i=1,2\right)$. At the present parameter values and amplitude scale, the terms $\alpha_{11}$ and $\alpha_{21}$ mainly affect the system dynamics by modifying the equivalent linear stiffness. In the amplitude-frequency response, they are reflected primarily as an overall shift in the resonance frequency, without significantly changing the shape of the response curve or the stability structure of the system, as shown in Figure 7a. Therefore, in practice, we can first calculate the center frequency using the formula of the linear system $\sqrt{\frac{\omega_{1}^{2}+\omega_{2}^{2}}{2}}$, and then shift to the left by $\alpha_{11}$ to obtain the actual center frequency. $\alpha_{11}$ varies with the gate bias as shown in Figure 7b. 

### 4.2. Bandwidth Enhancement in a Triple-Drum Coupled Resonator System

**Figure 8:**
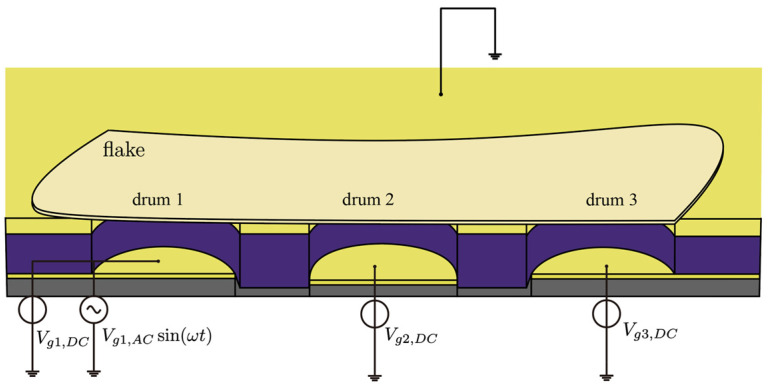
Schematic diagram of the three-resonator drum coupling structure.

We next extend the analysis to a three-drum coupled system, as shown in Figure 8. Here, Drum 1 acts as the driving drum, while Drums 2 and 3 serve as the readout drums. When an AC excitation signal of the μV level is superimposed on the gate DC bias of the driving drum, the multi-port filtering and signal selection functions can be achieved by separately detecting the amplitude responses of the two readout drums. Based on the structural model shown in Figure 8, the normalized coupling dynamic equation of the system can be written as:

(9) $$\left\{\begin{array}{c} {\ddot{x}}_{1}+\gamma_{1}{\dot{x}}_{1}+\omega_{1}^{2}x_{1}=\sum_{i=1}^{3}j_{1i}\cdot x_{c}^{i-1}\cdot x_{2}^{i}+\sum_{i=1}^{3}q_{1i}\cdot x_{c}^{i-1}\cdot x_{3}^{i}+\sum_{i=1}^{3}\alpha_{1i}\cdot x_{c}^{i-1}\cdot x_{1}^{i}+f_{d}\cos\left(\omega_{d}t\right)\\ {\ddot{x}}_{2}+\gamma_{2}{\dot{x}}_{2}+\omega_{2}^{2}x_{2}=\sum_{i=1}^{3}j_{2i}\cdot x_{c}^{i-1}\cdot x_{1}^{i}+\sum_{i=1}^{3}q_{2i}\cdot x_{c}^{i-1}\cdot x_{3}^{i}+\sum_{i=1}^{3}\alpha_{2i}\cdot x_{c}^{i-1}\cdot x_{2}^{i}\\ {\ddot{x}}_{3}+\gamma_{3}\dot{x_{3}}+\omega_{3}^{2}x_{3}=\sum_{i=1}^{3}j_{3i}\cdot x_{c}^{i-1}\cdot x_{1}^{i}+\sum_{i=1}^{3}q_{3i}\cdot x_{c}^{i-1}\cdot x_{2}^{i}+\sum_{i=1}^{3}\alpha_{3i}\cdot x_{c}^{i-1}\cdot x_{3}^{i} \end{array}\right.$$

where

(10) $$J_{1i}=J_{2i}=-\lambda^{2}\frac{V_{g,1}V_{g,2}}{2C_{m}^{\prime }}\frac{1}{\left(i+1\right)x_{c}^{i-1}}\left(i\geq 1\right)$$

(11) $$J_{3i}=q_{1i}=-\lambda^{2}\frac{V_{g,1}V_{g,3}}{2C_{m}^{\prime }}\frac{1}{\left(i+1\right)x_{c}^{i-1}}\left(i\geq 1\right)$$

(12) $$q_{2i}=q_{3i}=-\lambda^{2}\frac{V_{g,2}V_{g,3}}{2C_{m}^{\prime }}\frac{1}{\left(i+1\right)x_{c}^{i-1}}\left(i\geq 1\right)$$

(13) $$\alpha_{ki}=-\lambda^{2}\frac{V_{g,k}^{2}}{2C_{m}^{\prime }}\frac{1}{\left(i+1\right)x_{c}^{i-1}}\;\left(i\geq 1,k=1,2,3\right)$$

where $x_{k}\;\left(k=1,2,3\right)$ respectively represent the normalized displacements of the three drums, $\gamma_{k}$ and $\omega_{k}$ are the corresponding damping coefficients and natural frequencies. Here, $C_{m}^{\prime }$ is set to 0.15 pF, and the damping is adjusted to make 1/Q = 1.62 × $10^{-4}$; The coupling strengths still depend on the products of the corresponding DC gate biases, so by setting an appropriate gate bias, different filtering effects can be achieved in the two readout drums respectively.

**Figure 9:**
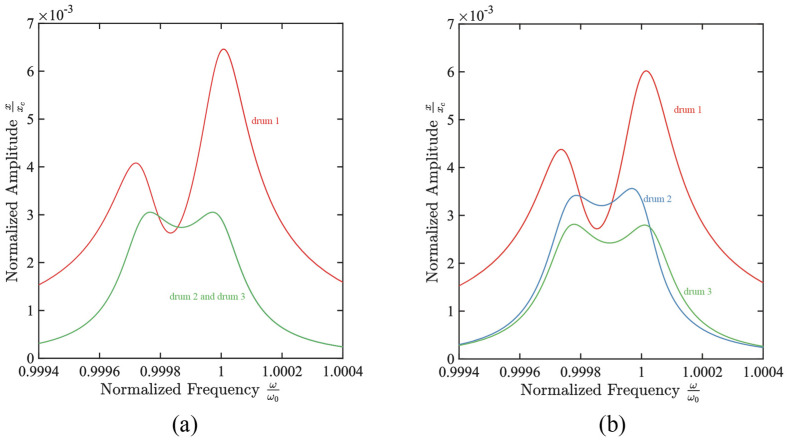
Numerically calculated frequency response of the three-resonator system under the selected gate-bias condition. (**a**) Frequency response curve under the condition of three-resonator drum coupling, when $V_{g1}=V_{g2}=V_{g3}=16\;V$, the system exhibits significant broadband filtering characteristics, with an effective bandwidth of 22.3 kHz, and the corresponding transition band slope and passband ripple are shown in the figure; (**b**) Changes in the frequency response of the three-drums system when a small gate bias perturbation $V_{g3}=16.0001\;V$ is applied to the readout drum 2 while keeping $V_{g1}=V_{g2}=16\;V$ unchanged.

As shown in Figure 9a, when the voltages of all three drums are 16 V, compared to the case of two drum coupling where $V_{g1}=V_{g2}=16\;V$, the bandwidth of 21.79 kHz is nearly four times larger than that in the case of two drums. The roll-off slope has changed to 5056.05 dB/MHz, and the ripple has become 0.95 dB. As shown in Figure 9b, when $V_{g1}=V_{g2}=16\;V$ and $V_{g3}=16.001\;V$ are maintained, the filter curve of drum 2 changes significantly, showing a gradual decrease in bandwidth and the roll-off slope changed to 4039.31 dB/MHz, with a bandwidth of 19.67 KHz, and the ripple decreased to 0.92 dB.

The significant bandwidth enhancement in the three-resonator drum system mainly comes from the formation of hybrid collective modes under strong coupling. In this scenario, the originally independent single drum eigenmodes are reorganized into multiple highly mixed coupling modes in both space and frequency, enabling the system to effectively receive and transmit external excitation energy over a wider frequency range, thereby significantly expanding the overall frequency response range. The effective bandwidth has clearly exceeded the inherent linewidth limited by the intrinsic damping of a single resonator.

Furthermore, it can be speculated that as the number of resonators participating in the coupling increases and the coupling strength strengthens, the number of modal channels available for energy exchange also increases, and the energy redistribution process between modes becomes more thorough, resulting in a wider response bandwidth in the frequency domain. However, it should be noted that the bandwidth expansion is not unlimited, and its specific form is constrained by the distribution of damping and the uneven participation degree of modes. Under strong coupling conditions, if the damping differences between different modes are significant, obvious passband fluctuations may occur in the system’s frequency response.

Therefore, by reasonably designing and matching the damping parameters of each resonator, it is possible to maintain the broadband response characteristics while effectively suppressing the amplitude fluctuations within the passband, thereby achieving a compromise optimization between bandwidth and flatness. This characteristic provides important design freedom for the application of multi-resonator coupling structures in broadband filtering and high-robustness frequency response devices.

## 5. Conclusions and Future Work

We propose and analyze a tunable NEMS filtering strategy based on two spatially separated MoS_2_ resonators. A nonlinear coupled model is developed, and bias-dependent expressions for the natural frequencies and coupling strength are derived, showing that the filter response is governed by the converged slow-flow steady state with a convergence time of ∼0.7 ms.

Two tuning strategies via gate DC biases ($x_{g}/x_{c}<0.1$) are examined: (i) fixing the frequency ratio and co-tuning both drums to shift the center frequency upward up to $∼1.5\times f_{0}$ (ii) keeping the coupling strength unchanged while fine-tuning the frequency ratio to achieve high-sensitivity bandwidth control (up to ∼200%) with essentially fixed center frequency; moreover, since $J\left(V_{g}\right)$ and $\omega \left(V_{g}\right)$ have different voltage sensitivities, appropriate choices of material and geometry can enable partial decoupling of J and ω during tuning. For a three-resonator system, strong coupling leads to collective mixed modes and additional energy-exchange channels, producing a much broader passband (e.g., ∼4× wider than the two-drum case at 16 V) while maintaining a steep transition and low ripple; under small detuning, the response remains nearly symmetric, and the nonlinear modulation mainly shifts the resonance via effective stiffness renormalization without substantially changing the response shape or stability. We also point out that within the current amplitude range, the nonlinear modulation effectively renormalizes the linear stiffness, producing a controllable resonance-frequency shift while leaving the amplitude–frequency response shape and stability essentially unchanged, which supports robust broadband filtering in multi-resonator designs.

In summary, the proposed coupled-resonator filtering strategy provides a tunable center frequency, a controllable bandwidth, and a fast, steady-state response. It should be noted that the present work is intended to demonstrate a feasible resonant filtering concept rather than a complete circuit-level filter implementation. Therefore, system-level metrics such as insertion loss and linear dynamic range are beyond the scope of the present study. Under the current model assumptions, the input AC signal corresponding to the considered operating regime is on the order of millivolts, which only reflects the theoretical signal scale rather than the actual achievable dynamic range of a practical device. The future work will involve achieving a higher degree of independent tuning by optimizing the geometric and capacitance parameters, as well as conducting detailed experimental implementation and system-level verification for the filter structure.

## Data Availability

The original contributions presented in this study are included in the article/Supplementary Material. Further inquiries can be directed to the corresponding authors.

## Supplementary Materials

The following supporting information can be downloaded at: https://www.mdpi.com/article/10.3390/mi17030379/s1. References [26,27] are cited in Supplementary File.

## Abbreviations

The following abbreviations are used in this manuscript:

N/MEMS	Micro/Nanoelectromechanical Systems
2D	Two-Dimensional
RF	Radio Frequency
IoT	Internet of Things
MBAN	Medical Body Area Network
SDR	Software-Defined Radio
VCTs	Vibrating Channel Transistors
