# Supplementary material for: Tunable Bandpass Filtering in Coupled Nanodrums Enabled by 1:1 Internal Resonance
Source: Micromachines (Basel). 2026 Mar 20;17(3):379. doi: 10.3390/mi17030379 (PMC13028577; doi:10.3390/mi17030379)
Supplement: Supplementary file 1 [file micromachines-17-00379-s001.zip › micromachines-4206070-supplementary.pdf]

Supplementary S1 Electromechanical Coupling Model

Supplementary S1.1 Preliminaries

Using a single-mode approximation for the membrane deflection, as commonly adopted in Refs. [26,27],

$$\xi(a) = x_g\left(1 - \frac{a^2}{r^2}\right), \quad a \in [0, r],$$

the capacitance can be written as

$$C[x_1(t)] = \int_0^{r_g} \frac{\varepsilon_0\, 2\pi a\, \mathrm{d}a}{x_c - (x_g + x)\phi(a)}.$$

Since

$$\left|\frac{x_g\phi(a) + x\,\phi(a)}{x_c}\right| < 1,$$

a geometric-series expansion can be applied:

$$\frac{1}{x_c - x_g\phi - x\phi} = \frac{1}{x_c}\sum_{n=0}^{\infty}\left(\frac{(x_g + x)\,\phi(a)}{x_c}\right)^n.$$

Noting that

$$\phi(a)^2 = 1 - \frac{2a^2}{r^2} + \frac{a^4}{r^4}, \qquad \phi(a)^3 = 1 - \frac{3a^2}{r^2} + \frac{3a^4}{r^4} - \frac{a^6}{r^6},$$

for $r_g = r$ we have

$$\begin{aligned} I_n &= \int_0^r a\ \phi(a)^n\,\mathrm{d}a \\ &= \int_0^r a\left(1 - \frac{a^2}{r^2}\right)^n \mathrm{d}a \\ &= \frac{r^2}{2(n+1)}. \end{aligned}$$

Substituting gives

$$C(x) = \varepsilon_0 2\pi \sum_{n=0}^{\infty} \frac{(x_g + x)^n}{x_c^{n+1}} \int_0^{r_g} a\ \phi(a)^n\,\mathrm{d}a$$

$$= \varepsilon_0 2\pi \left[\frac{r^2}{2x_c} + \frac{(x_g + x)r^2}{4x_c^2} + \frac{(x_g + x)^2 r^2}{6x_c^3} + \frac{(x_g + x)^3 r^2}{8x_c^4} + \cdots \right]$$

$$= \frac{\varepsilon_0 \pi r^2}{x_c} \left[1 + \frac{(x_g + x)}{2x_c} + \frac{(x_g + x)^2}{3x_c^2} + \cdots \right].$$

Since $r_g = r$, we denote the time-varying part of C_1 by $C_{1,\mathrm{AC}}$. Keeping terms up to cubic order, we have

$$C_{1,\mathrm{AC}}(t) \approx \frac{\varepsilon_0 \pi r^2}{x_c^2} \left(\frac{x_1(t)}{2} + \frac{x_1(t)^2 - x_{10}^2/2}{3x_c} + \frac{x_1(t)^3}{4x_c^2} \right).$$

Here $x_1(t) = x_{10}\sin(\omega_d t)$. For convenience, we define $\lambda = \frac{\varepsilon_0 \pi r^2}{x_c^2}$.

Supplementary S1.2 Derivation of *J*

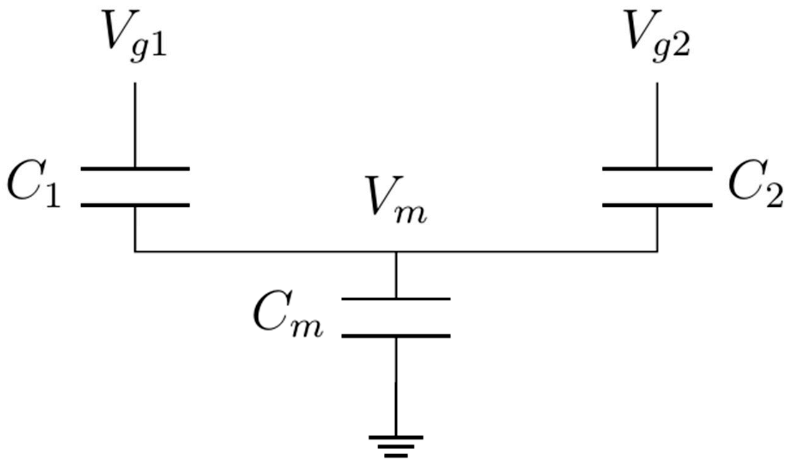

Fig1:Equivalent circuit schematic.

Here, C_1 and C_2 denote the gate-to-membrane capacitances of the drive and sense resonators, respectively, and C_m denotes the parasitic capacitance between the membrane and the top electrode. Let the parasitic capacitance between the membrane and the top electrode be $C_m = 0.468$ pF. The free charge on the membrane-connected plate of the drive resonator is

$$Q_1 = (C_1 + C_{1,\mathrm{AC}})(V_{g1} + V_{g1,\mathrm{AC}} - V_m - V_{m,\mathrm{AC}}).$$

The free charge on the membrane-connected plate of the sense resonator is

$$Q_2 = (C_2 + C_{2,\mathrm{AC}})(V_{g2} - V_m - V_{m,\mathrm{AC}}); \quad V_{g2,\mathrm{AC}} \approx 0.$$

The free charge on the parasitic capacitor plate connected to the membrane is

$$Q_m = (C_m + C_{m,\text{AC}})(0 - V_m - V_{m,\text{AC}}).$$

Supplementary S1.2.1 DC grounded, AC floating

In the DC case, the parasitic capacitor C_m is absent; the membrane potential is directly set by the resistive path to ground. For small-signal AC analysis, we set the DC quantities to zero. The charge-neutrality condition gives

$$C_1(V_{g1,\text{AC}} - V_{m,\text{AC}}) + C_{1,\text{AC}}(V_{g1} - V_m) - C_2 V_{m,\text{AC}} + C_{2,\text{AC}}(V_{g2} - V_m) - C_m V_{m,\text{AC}} - C_{m,\text{AC}} V_m = 0.$$

Solving for $V_{m,\text{AC}}(t)$ yields

$$V_{m,\text{AC}}(t) = \frac{C_1 V_{g1,\text{AC}}(t) + C_{1,\text{AC}}(V_{g1} - V_m) + C_{2,\text{AC}}(V_{g2} - V_m) - C_{m,\text{AC}} V_m}{C_1 + C_2 + C_m}.$$

Under the condition $C_m \gg C_{1,2}$, and the membrane is grounded through the top electrode, i.e., $V_m = 0$, we obtain the approximation

$$V_{m,\text{AC}}(t) \approx \frac{C_1 V_{g1,\text{AC}} + C_{1,\text{AC}} V_{g1} + C_{2,\text{AC}} V_{g2}}{C_m}.$$

Moreover,

$$\frac{C_{1,\text{AC}}\, V_{g1}}{C_1\, V_{g1,\text{AC}}} = \frac{x_1}{x_c} \frac{V_{g1}}{V_{g1,\text{AC}}} \sim 10^2,$$

and therefore

$$V_{m,\text{AC}}(t) \approx \frac{C_{1,\text{AC}} V_{g1} + C_{2,\text{AC}} V_{g2}}{C_m}.$$

A variation in $V_{m,\text{AC}}$ modifies the electrostatic force acting on the passive resonator[1]. Hence,

$$F_2(x) = \frac{1}{2} \frac{\partial C}{\partial x} V^2 = \frac{1}{2} \left(V_{g2} - V_m - V_{m,\text{AC}}\right)^2 \frac{dC_{2,\text{AC}}}{dx}.$$

Equivalently,

$$F_2(x) = \frac{1}{2} \left(V_{g2} - V_m - V_{m,\text{AC}}\right)^2 \frac{\varepsilon_0 \pi r^2}{x_c^2} \left(\frac{1}{2} + \frac{2x_2(t)}{3x_c} + \frac{3x_2(t)^2}{4x_c^2}\right).$$

---

[1] $U = \frac{1}{2} C V^2$, and differentiating with respect to the gap coordinate yields the electrostatic force for a parallel-plate capacitor.

Therefore,

$$F_{2,\mathrm{AC}}(t)=\frac{1}{2}\left(-2V_{g2}V_{m,\mathrm{AC}}+2V_mV_{m,\mathrm{AC}}\right)\frac{\varepsilon_0\pi r^2}{x_c^2}\left(\frac{1}{2}+\frac{2x_2(t)}{3x_c}+\frac{3x_2(t)^2}{4x_c^2}\right).$$

Since $V_m=0$ and $x_1\ll x_c$, we simplify to

$$F_{2,\mathrm{AC}}(t)=-V_{g2}V_{m,\mathrm{AC}}\frac{\varepsilon_0\pi r^2}{2x_c^2}.$$

Substituting $V_{m,\mathrm{AC}}(t)$ gives

$$F_{2,\mathrm{AC}}(t)=-\frac{\lambda^2}{2C_m}\left[\left(\frac{x_1(t)}{2}+\frac{x_1(t)^2}{3x_c}+\frac{x_1(t)^3}{4x_c^2}\right)V_{g1}V_{g2}+\left(\frac{x_2(t)}{2}+\frac{x_2(t)^2}{3x_c}+\frac{x_2(t)^3}{4x_c^2}\right)V_{g2}^2\right].$$

Thus,

$$\boxed{J_{2i}=-\lambda^2\frac{V_{g,1}V_{g,2}}{2C_m}\frac{1}{(i+1)x_c^{i-1}}\quad(i\geq 1)}.$$

The nonlinear spring (self-nonlinearity) terms are

$$\boxed{\alpha_{2i}=-\lambda^2\frac{V_{g,2}^2}{2C_m}\frac{1}{(i+1)x_c^{i-1}}\quad(i\geq 1)}.$$

Supplementary S1.3 Normalization of the governing equations

First, note the original model

$$\boxed{\begin{cases}\ddot{x}_1+\gamma_1\dot{x}_1+\omega_1^2x_1 & =j_{11}x_2+j_{12}x_2^2+j_{13}x_2^3+\alpha_{11}x_1+\alpha_{12}x_1^2+\alpha_{13}x_1^3+f_d\cos(\omega_d t),\\ \ddot{x}_2+\gamma_2\dot{x}_2+\omega_2^2x_2 & =j_{21}x_1+j_{22}x_1^2+j_{23}x_1^3+\alpha_{21}x_2+\alpha_{22}x_2^2+\alpha_{23}x_2^3.\end{cases}}$$

We introduce the nondimensional time $\tilde{t}=\Omega t$ .Then $\ddot{\tilde{x}}=(1/\Omega^2)\ddot{x}$, $\dot{\tilde{x}}=(1/\Omega)\dot{x}$, $\widetilde{x_\iota}=x_i$, and $\widetilde{\omega_\iota}=\frac{\omega_i}{\Omega}$[2] Therefore, Eq. (15) becomes

$$\begin{cases}\ddot{x}_1+\frac{\gamma_1}{\Omega}\dot{x}_1+\left(\frac{\omega_1}{\Omega}\right)^2x_1 & =\frac{j_{11}}{\Omega^2}x_2+\frac{j_{12}}{\Omega^2}x_2^2+\frac{j_{13}}{\Omega^2}x_2^3+\frac{\alpha_{11}}{\Omega^2}x_1+\frac{\alpha_{12}}{\Omega^2}x_1^2+\frac{\alpha_{13}}{\Omega^2}x_1^3+\frac{\widetilde{f_d}}{\Omega^2}\cos\left(\frac{\omega_d}{\Omega}t\right),\\ \ddot{x}_2+\frac{\gamma_2}{\Omega}\dot{x}_2+\left(\frac{\omega_2}{\Omega}\right)^2x_2 & =\frac{j_{21}}{\Omega^2}x_1+\frac{j_{22}}{\Omega^2}x_1^2+\frac{j_{23}}{\Omega^2}x_1^3+\frac{\alpha_{21}}{\Omega^2}x_2+\frac{\alpha_{22}}{\Omega^2}x_2^2+\frac{\alpha_{23}}{\Omega^2}x_2^3\end{cases}$$

We further introduce the normalized displacement $\hat{x}=\frac{x}{x_c}$, and the original formula is simplified to

---

[2] Here $\tilde{T}=\Omega T$.

$$\begin{cases} \ddot{x}_1 + \frac{\gamma_1}{\Omega}\dot{x}_1 + \left(\frac{\omega_1}{\Omega}\right)^2 x_1 & = \frac{j_{11}}{\Omega^2}x_2 + x_c\frac{j_{12}}{\Omega^2}x_2^2 + x_c^2\frac{j_{13}}{\Omega^2}x_2^3 + \frac{\alpha_{11}}{\Omega^2}x_1 + x_c\frac{\alpha_{12}}{\Omega^2}x_1^2 + x_c^2\frac{\alpha_{13}}{\Omega^2}x_1^3 + \frac{\widetilde{f_d}}{\Omega^2 x_c}\cos\left(\frac{\omega_d}{\Omega}t\right), \\ \ddot{x}_2 + \frac{\gamma_2}{\Omega}\dot{x}_2 + \left(\frac{\omega_2}{\Omega}\right)^2 x_2 & = \frac{j_{21}}{\Omega^2}x_1 + x_c\frac{j_{22}}{\Omega^2}x_1^2 + x_c^2\frac{j_{23}}{\Omega^2}x_1^3 + \frac{\alpha_{21}}{\Omega^2}x_2 + x_c\frac{\alpha_{22}}{\Omega^2}x_2^2 + x_c^2\frac{\alpha_{23}}{\Omega^2}x_2^3 \end{cases}$$

Supplementary S2 Computation of the Effective Stiffness k

We consider a circular monolayer membrane with radius $r = 2\,\mu$m and thickness $h = 0.65$ nm, Young's modulus $E = 270$ GPa, Poisson's ratio $\nu = 0.27$, volumetric mass density $\rho = 5060\ \mathrm{kg/m^3}$, and pre-strain $\varepsilon_0 = 1.0 \times 10^{-3}$. The initial gate gap is $x_c = 300$ nm, and an electrostatic DC bias V_g is applied.

Supplementary S2.1 Membrane model

For a monolayer membrane, assuming the first mode dominates, we adopt the fundamental-mode shape

$$\xi(a) = x\left(1 - \frac{a^2}{r^2}\right), \qquad a \in [0, r],$$

where $x = \xi(0)$ and $\xi(r) = 0$.

Supplementary S2.2 System potential energy

When $D \ll \sigma_0 r^2$, the stretching energy $\gg$ the bending energy, and the elastic potential energy can be written as

$$U_{\mathrm{el}}(x) = \frac{\pi E h}{1 - \nu^2}\left(\frac{2x^4}{3r^2} + \varepsilon_0 x^2 + \frac{1}{2}\varepsilon_0^2 r^2\right).$$

If the membrane–gate separation is x_c and the membrane deflection is $\xi(a)$, then

$$C[\xi] \;\approx\; \int \frac{\varepsilon_0}{x_c - \xi(a)}\,\mathrm{d}A,$$

where

$$\frac{1}{x_c - \xi} \approx \frac{1}{x_c}\left(1 + \frac{\xi}{x_c} + \frac{\xi^2}{x_c^2} + \cdots\right).$$

With the previous assumption $\xi(a) = x\phi(a)$, and under a consistent approximation order, we obtain

$$C(x) = \frac{\varepsilon_0 \pi r_g^2}{x_c - x}.$$

Therefore, the electrostatic potential energy is

$$U_{\mathrm{es}}(x) = -\frac{1}{2} C(x) V_g^2.$$

Supplementary S2.3 Equilibrium deflection x_g

The equilibrium condition is given by setting the first derivative of the total potential energy with respect to x to zero:

$$\frac{\partial(U_{\mathrm{el}}+U_{\mathrm{es}})}{\partial x}=0.$$

Substituting

$$C(x)=\frac{\varepsilon_0\pi r^2}{x_c}\left[1+\frac{x}{2x_c}+\frac{x^2}{3x_c^2}+\cdots\right]$$

leads to the nonlinear equation

$$\frac{\pi Eh}{1-\nu^2}\left(\frac{8x_g^3}{3r^2}+2\varepsilon_0 x_g\right)-\frac{V_g^2\varepsilon_0\pi r^2}{2x_c^2}\left[\frac{1}{2}+\frac{2x_g}{3x_c}+\cdots\right]=0.$$

For each applied gate voltage V_g, the corresponding equilibrium deflection x_g is obtained by numerically solving Eq. [xg_eq] in MATLAB 2025b.

Supplementary S2.4 Effective stiffness k

Taking the second derivative of the total potential energy at the equilibrium point yields

$$\begin{aligned} k &= \left.\frac{\partial^2(U_{\mathrm{el}}+U_{\mathrm{es}})}{\partial x^2}\right|_{x=x_g} \\ &= \frac{\pi Eh}{1-\nu^2}\left(\frac{8x_g^2}{r^2}+2\varepsilon_0\right)-\frac{\varepsilon_0\pi r^2 V_g^2}{3x_c^3}. \end{aligned}$$

The first term corresponds to the geometric and pre-strain stiffness, and the second term is the electrostatic softening contribution (negative sign).

$$\boxed{\alpha=\frac{1}{2}\frac{\partial^3 U_{\mathrm{el}}}{\partial x^3}|_{x_g}=\frac{1}{2}\frac{96\pi Eh}{(1-\nu^2)r^2}x_g}$$

$$\boxed{\beta=\frac{1}{6}\frac{\partial^4 U_{\mathrm{el}}}{\partial x^4}=\frac{16\pi Eh}{(1-\nu^2)r^2}}$$

Supplementary S2.5 Computation of the Effective Mass m_{eff}

A monolayer membrane can be approximated as a tension-dominated system; its effective mass is taken as the fundamental-mode coefficient $\alpha\approx 0.269$

$$m_{\mathrm{eff}}=\alpha\,\rho h\pi r^2,\qquad \alpha\approx 0.269.$$

Supplementary S2.6 Natural Frequency of a Monolayer Membrane ω_0

The natural angular frequency is

$$\boxed{\omega_0 = \sqrt{\frac{k}{m_{\mathrm{eff}}}} = \sqrt{\frac{\frac{\pi E h}{1-\nu^2}\left(\frac{8x_g^2}{r^2} + 2\varepsilon_0\right) - \frac{\varepsilon_0 \pi r_g^2 V_g^2}{\left(x_c - x_g\right)^3}}{\alpha \rho h \pi r^2}}.}$$

The variation of the natural frequency ω_0 with gate voltage V_g, calculated from Eq. (24), is presented in Fig. 2(c) of the main text.

Supplementary S3 Derivation of the Center Frequency for a Linear Two-Drum Coupled System

Consider the following two-degree-of-freedom linear coupled oscillator system:

$$\begin{cases} \ddot{x}_1 + \gamma_1 \dot{x}_1 + \omega_1^2 x_1 = j x_2 + f_d \cos \omega_d t, \\ \ddot{x}_2 + \gamma_2 \dot{x}_2 + \omega_2^2 x_2 = j x_1. \end{cases}$$

To derive the natural frequencies and their center value, we neglect damping and external forcing and retain only the free-vibration part:

$$\begin{cases} \ddot{x}_1 + \omega_1^2 x_1 = j x_2, \\ \ddot{x}_2 + \omega_2^2 x_2 = j x_1. \end{cases}$$

Supplementary S3.1 Assume harmonic solutions and form the characteristic equation

Assume harmonic motion of the two drums:

$$x_1(t) = A_1 e^{i\omega t}, \qquad x_2(t) = A_2 e^{i\omega t}.$$

Substituting into [eq:free] gives

$$\begin{pmatrix} -\omega^2 + \omega_1^2 & -j \\ -j & -\omega^2 + \omega_2^2 \end{pmatrix} \begin{pmatrix} A_1 \\ A_2 \end{pmatrix} = \begin{pmatrix} 0 \\ 0 \end{pmatrix}.$$

For a nontrivial solution to exist, the determinant of the coefficient matrix must vanish:

$$(-\omega^2 + \omega_1^2)(-\omega^2 + \omega_2^2) - j^2 = 0.$$

Supplementary S3.2 Rewrite as a quadratic equation in ω^2

Let $y = \omega^2$. Equation [eq:det] becomes

$$(y - \omega_1^2)(y - \omega_2^2) - j^2 = 0.$$

Expanding yields

$$y^2 - (\omega_1^2 + \omega_2^2) y + (\omega_1^2 \omega_2^2 - j^2) = 0.$$

The two roots (i.e., the squared natural frequencies of the two modes) are

$$\omega_\pm^2 = \frac{\omega_1^2 + \omega_2^2 \pm \sqrt{(\omega_1^2 - \omega_2^2)^2 + 4j^2}}{2}.$$

Supplementary S3.3 Center frequency

Define the center frequency (the midpoint between the two modal locations) as the average of the squared modal frequencies:

$$\omega_0^2 = \frac{\omega_+^2 + \omega_-^2}{2}.$$

From the coefficient relations of the quadratic equation,

$$\omega_+^2 + \omega_-^2 = \omega_1^2 + \omega_2^2,$$

and hence

$$\boxed{\omega_0^2 = \frac{\omega_1^2 + \omega_2^2}{2}}.$$

Therefore, the center frequency is

$$\boxed{\omega_0 = \sqrt{\frac{\omega_1^2 + \omega_2^2}{2}}}.$$

For brevity, we introduce the *mode splitting* quantity

$$\Delta = \sqrt{(\omega_1^2 - \omega_2^2)^2 + 4j^2},$$

so that

$$\omega_\pm^2 = \omega_0^2 \pm \frac{\Delta}{2}.$$

In the nearly degenerate case where the two drums have almost equal natural frequencies ($\omega_1 \approx \omega_2 \approx \omega_0$), we have $\omega_1^2 - \omega_2^2 \approx 0$ and $\Delta \approx 2|j|$, hence

$$\omega_\pm^2 \approx \omega_0^2 \pm |j|, \qquad \Rightarrow \qquad \omega_\pm \approx \omega_0 \pm \frac{|j|}{2\omega_0}.$$
